# Responding to COVID-19 in Brunei Darussalam: Lessons for small countries

**DOI:** 10.7189/jogh.10.010363

**Published:** 2020-06

**Authors:** Justin Wong, Wee Chian Koh, Mohammad Fathi Alikhan, Anita B Z Abdul Aziz, Lin Naing

**Affiliations:** 1 Disease Control Division, Ministry of Health, Brunei Darussalam; 2 Centre for Strategic and Policy Studies, Brunei Darussalam; 3 Universiti Brunei Darussalam, Brunei Darussalam; 4 PAPRSB Institute of Health Sciences, Universiti Brunei Darussalam, Brunei Darussalam

On January 30, 2020, the World Health Organization (WHO) declared Coronavirus Disease 2019 (COVID-19) a “Public Health Emergency of International Concern”, and the disease now affects almost all countries and areas. While much of the discussion around pandemic preparedness and response has centered around larger countries or those with advanced economies, small countries face different and specific challenges in responding to this event [1]. For many small countries, where the financial envelope is necessarily smaller, pandemic preparedness may rank lower down the list of other more immediate health priorities [2].

Brunei Darussalam, a country with a population of 459 400, recorded its first imported case on March 9, and as of April 20 has detected 138 cases. Assessed against several parameters including a slowing trajectory since the 100^th^ confirmed case, limited local transmission, and the absence of cases with no known epidemiological links, Brunei compares favorably with Singapore, Taiwan, and others considered to have implemented a successful response operation.

Despite early success, Brunei must prepare for the possibility of sustained community transmission given the escalating global situation [3]. While the country has advantages including its relative wealth, a very high human development index, and universal health coverage, this scenario presents a specific set of challenges for small countries like Brunei (**Table 1**). We review Brunei’s response across three thematic areas and propose lessons for other small countries.

**Table 1:** Challenges and opportunities of small countries in preparing for community transmission of COVID-19

Characteristics	Challenges	Opportunities
Multiple land borders and high connectivity to other countries	Vulnerable to multiple importation events	Implementation of proportionate measures at point of entry
	Export of disease to other countries	
	Travel and trade restrictions	Collaboration with neighbouring countries for rapid exchange of information and joint risk assessment
Lack of state capacity or prior experience in managing large outbreaks or natural disasters	Infrequent activation of existing coordination mechanisms may result in over-reliance on strong interpersonal relationships for multi-agency working	Ensure rationalisation of managerial responsibilities in crisis
	Institutional memory and resilience are more fragile	Clarity on accountability for different plans and procedures, supplemented by documentation of processes to reinforce resilience
Limited local health workforce and health service facilities	Healthcare services may quickly become overwhelmed with demands for critical care beds and other equipment	Health workforce can be redeployed in early phases of the epidemic to regain control
	Other non-COVID-19 health services may be neglected	Contact tracing and quarantine measures can slow down epidemic progression
Reliance on imports for PPE and essential medical supplies	Supply chain disruptions and travel restrictions may limit availability of essential medical supplies to effectively manage the pandemic	PPE and essential drug stockpiling during ‘*peacetime*’ can be augmented by regional collaboration and strong bilateral and multilateral relationships to ensure supply lines remain constant
Information tends to spread quickly in smaller communities with multiple information sources	False information may spread quickly and cause public panic, which can be difficult to control on unconventional platforms or social media	Ensuring transparency and openness of information from health authorities that are responsive to public concerns
Heterogeneous population and significant foreign worker population	Different cultural expectations surrounding social distancing measures	Engagement with community and religious leaders for targeted approaches
	Some groups such as foreign workers may be harder to reach	Foreign missions can provide assistance in ensuring foreign worker access to health care

PPE – personal protective equipment

## INCIDENT MANAGEMENT, PLANNING, AND MULTI-SECTORAL COORDINATION

Brunei’s government is highly centralized and there is a dedicated budget allocation of BND15 million (US$10.5 million) for outbreaks and public health emergencies. Officially, oversight for the COVID-19 response is with the National Disaster Council, a multi-agency group, although the council has convened only occasionally. The Ministry of Health Emergency Operations Centre manages day-to-day issues with ad hoc support from other agencies. However, the lack of an updated resource map and resource-pooling arrangements hampered initial efforts. Moreover, there is a lack of clarity on responsibilities for securing operational logistic arrangements.

Many small countries lack experience in crisis management, and response capacities are often untested in real-life events [4]. Although Brunei has a range of legislation that support crisis management, it lacks experience in handling large-scale outbreaks due to infrequent activation of existing coordination mechanisms. Yet strong interpersonal relationships can mitigate the lack of more formal structures, as evident in Brunei’s ad hoc multi-agency arrangements at the technical and operational level. Leveraging these informal networks, at least in the initial phases, can allow for more formal mechanisms to emerge naturally over the course of the outbreak. This should be supplemented by efforts to establish clear lines of accountability and documentation of processes to preserve institutional memory.

## SURVEILLANCE AND LABORATORY TESTING

Enhanced surveillance mechanisms in place since January meant that in the course of the investigation of its first case, Brunei was able to recognize and alert the global health community of an international super-spreading event – the Tablighi Jamaat cluster – the first country to do so. An estimated 15 000 participants from around the world had attended a religious event in Malaysia in late February, including 81 from Brunei. 19 infected locals returned home and transmitted the disease to family members, coworkers, and many others in a local religious gathering, marking the start of Brunei’s first COVID-19 cluster [5]. Brunei’s success in controlling this first wave owes much to its surveillance mechanism supported by rigorous contact tracing. The surveillance system leverages on digital patient records in the national health information management system database that links all health care facilities with near 100% penetration of the population. There are dedicated teams for contact tracing comprising trained public health officers and field workers. Contact tracing is done manually which can be time and resource intensive [2]. In order to ensure sustainability of contact tracing activities, the contact tracing teams are supplemented by police officers who assist in case investigation and activity mapping.

**Figure:**
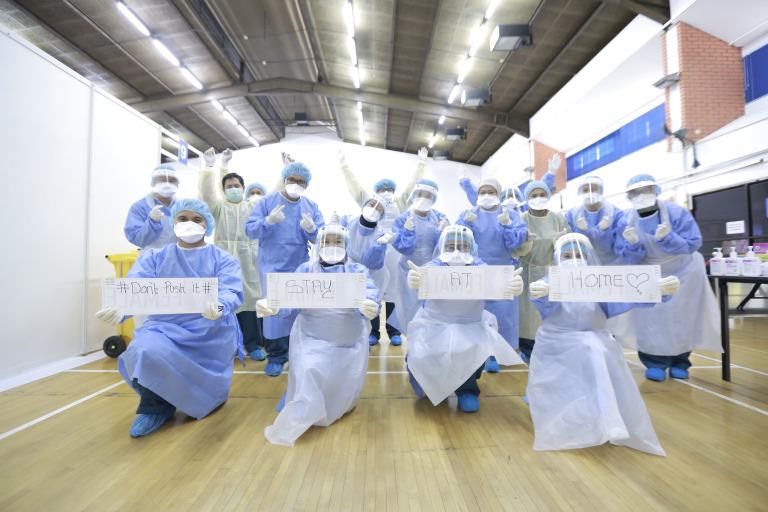
Photo: From the collection of Mas Fahri Azahari, used with permission.

Brunei is an early adopter of RT-PCR testing in the absence of symptoms. COVID-19 testing is mandatory under the Infectious Disease Act for all individuals with (i) travel or close-contact history (regardless of symptoms); (ii) pneumonia; or (ii) present a second time at a health care facility for an influenza-like illness within a 14-day period. In addition, random sampling is also conducted in community health centers, and among the large foreign worker population.

To support this considerable testing strategy, a sports complex has been converted into a 24-hour testing facility and a new Molecular Diagnostic Unit for Respiratory Viruses (built in three weeks) has begun operations, increasing *SARS-CoV-2* testing capacity from 240 to 1000 specimens per day [6]. As of April 20, Brunei’s test-per-capita ratio is 2479 in 100 000, among the world’s highest.

Brunei’s small population size and centralized government enabled rapid implementation of an enhanced surveillance mechanism and a mass testing program. We propose this model for other small countries, especially those with limited critical care infrastructure and surge capacity. Containment efforts can be crucial in delaying the onset of widespread community transmission, buying vital time to prepare mitigation measures and build capacity for supporting later stages of the pandemic.

## RISK COMMUNICATION

A significant public communications strategy was established, and the government has been more transparent and responsive than usual. A unique (for Brunei) feature of the response are daily press conferences broadcast live on national television and social media channels. These are led by the Health Minister who is frequently joined by other ministers to address queries from the press and public. A dedicated 24-hour hotline for public inquiries and a self-screening mobile application integrated with artificial intelligence and data analytic capabilities have also been established [7]. In general, this transparency has won public trust. Civic society has also played a key role, which is uncommon in the country’s top-down governance structure. A significant grassroots movement sparked a surge of volunteerism and community advocacy to reinforce the official government stance on social distancing and personal hygiene [8].

We recommend that small countries capitalize on their relative ease of information dissemination and invest in risk communication activities that engage the whole society. In Brunei, leadership within the health sector was visible, supported by other sectors, and seen to be responsive to public concerns. Engagement with both traditional and non-traditional media such as social media influencers and local personalities have paid off with momentum generated by local grassroot efforts serving as a counterweight to small pockets of misinformation on social media. At later stages of the pandemic, governments may need communities to sacrifice social well-being and job security during periods of enhanced social distancing. Initial efforts at gaining public trust and support will be crucial in securing compliance to these measures and sustaining the response [9].

Looking ahead, as *SARS-CoV-2* is expected to remain circulating, planning an exit strategy that minimises socioeconomic damage and accounts for potential disease resurgence should be initiated [10]. Adopting a phased approach to de-escalation, including gradual relaxation of social distancing measures and leveraging technology for swift contact tracing, could be a viable strategy to sustain the response to the COVID-19 pandemic [11].
